# Effect of temperature and time delay in centrifugation on stability of select biomarkers of nutrition and non-communicable diseases in blood samples

**DOI:** 10.11613/BM.2019.020708

**Published:** 2019-06-15

**Authors:** Ransi Ann Abraham, Praween K. Agrawal, Rajib Acharya, Avina Sarna, Sowmya Ramesh, Robert Johnston, Arjan de Wagt, Nizamuddin Khan, Akash Porwal, Sucheta Banerjee Kurundkar, Arvind Pandey, Raghu Pullakhandam, Krishnapillai Madhavan Nair, Geeta Trilok Kumar, H P S Sachdev, Umesh Kapil, Renu Saxena, Sila Deb, Ajay Khera, Lakshmy Ramakrishnan

**Affiliations:** 1 Department of Cardiac Biochemistry, All India Institute of Medical Sciences, Delhi, India; 2 UNICEF, Delhi, India; 3 Population Council, Delhi, India; 4 Clinical Development Services Agency, Translational Health Science & Technology Institute, Faridabad, Haryana; 5 Ex Director, National Institute of Medical Statistics, Indian Council of Medical Research, Delhi, India; 6 National Institute of Nutrition, Hyderabad, Telangana, India; 7 Institute of Home economics, Delhi, India; 8 Senior Consultant, Paediatrics and Clinical Epidemiology, B-16 Qutab Institutional Area, New Delhi, India; 9 Department of Human Nutrition, All India Institute of Medical Sciences, Delhi, India; 10 Department of Hematology, All India Institute of Medical Sciences, Delhi, India; 11 Ministry of Health and Family Welfare, Delhi, India

**Keywords:** survey, preanalytical conditions, temperature, time delay, centrifugation

## Abstract

**Introduction:**

Preanalytical conditions are critical for blood sample integrity and poses challenge in surveys involving biochemical measurements. A cross sectional study was conducted to assess the stability of select biomarkers at conditions that mimic field situations in surveys.

**Material and methods:**

Blood from 420 volunteers was exposed to 2 – 8 °C, room temperature (RT), 22 – 30 °C and > 30 °C for 30 min, 6 hours, 12 hours and 24 hours prior to centrifugation. After different exposures, whole blood (N = 35) was used to assess stability of haemoglobin, HbA1c and erythrocyte folate; serum (N = 35) for assessing stability of ferritin, C-reactive protein (CRP), vitamins B12, A and D, zinc, soluble transferrin receptor (sTfR), total cholesterol, high density lipoprotein cholesterol (HDL), low density lipoprotein cholesterol (LDL), tryglicerides, albumin, total protein and creatinine; and plasma (N = 35) was used for glucose. The mean % deviation of the analytes was compared with the total change limit (TCL), computed from analytical and intra-individual imprecision. Values that were within the TCL were deemed to be stable.

**Result:**

Creatinine (mean % deviation 14.6, TCL 5.9), haemoglobin (16.4%, TCL 4.4) and folate (33.6%, TCL 22.6) were unstable after 12 hours at 22-30°C, a temperature at which other analytes were stable. Creatinine was unstable even at RT for 12 hours (mean % deviation: 10.4). Albumin, CRP, glucose, cholesterol, LDL, triglycerides, vitamins B12 and A, sTfR and HbA1c were stable at all studied conditions.

**Conclusion:**

All analytes other than creatinine, folate and haemoglobin can be reliably estimated in blood samples exposed to 22-30°C for 12 hours in community-based studies.

## Introduction

India faces a unique challenge of under and over nutrition among general population, particularly in children (*1*). To obtain reliable estimate of the burden, a nationally representative survey, Comprehensive National Nutrition Survey (CNNS) is being undertaken in 0-19 years old children and adolescents. The survey includes analysis of biochemical indicators of nutrition, which are more objective measures as compared to questionnaire-based assessments, which are semi quantitative. The objective of CNNS is to assess the national prevalence of important micronutrient deficiencies, subclinical inflammation and cardiovascular risk through the measurement of biomarkers. The survey aims to measure circulating concentrations of haemoglobin, ferritin, C-reactive protein (CRP), vitamin B12, vitamin A (retinol), vitamin D, zinc, folate, soluble transferrin receptor (sTfR), glucose, glycated haemoglobin (HbA1c), total cholesterol, high density lipoprotein cholesterol (HDL), low density lipoprotein cholesterol (LDL), triglycerides (TG), albumin, total protein and creatinine in approximately 50,000 children and adolescents.

Reliable biochemical assessment depends on preanalytical, analytical and postanalytical conditions among which the preanalytical stage that includes blood collection is the most vulnerable to errors. Its management is therefore of paramount importance. Large scale surveys such as CNNS needs to focus on minimizing preanalytical variations through ensuring appropriate subject preparation and identification, proper sample collection, processing, transporting and storage. India due to its vast geography and climatic conditions faces unique challenges in terms of minimizing preanalytical variations in surveys. Due to regional and remote sample collection locations and lack of access to centrifugation, exposure of the specimen to variable temperatures prior to separation of serum/plasma is likely to occur. Temperature and time between collection and testing are critical for sample integrity (*2*). Pre-analytical variations arising due to differences in sample handling conditions may alter the properties of the analytes being measured. Although it is ideal to maintain the pre-analytical conditions uniformly, this is not always feasible in large scale surveys. Therefore, stability of biomarkers needs to be ensured across variety of anticipated pre-analytical conditions encountered in field settings, particularly the temperature and time taken for centrifugation.

There are very few studies on the stability of analytes in whole blood prior to centrifugation (*3*-*6*). Dupuy *et al*. in a recent study examined the stability of 35 analytes in blood samples kept for different lengths of time prior to centrifugation and reported most analytes to be stable in whole blood kept for 24 hours (*7*). Similarly, Oddoze *et al.* examined the stability of 81 analytes in whole blood at different temperatures for up to 72 hours and reported that most analytes were stable upto 24 hours (*6*). Nevertheless, most reported studies have done collection of blood in the laboratory and do not represent the true operational conditions of large scale field surveys, as transportation of samples to the analytical laboratory could be a potential variable. Thus, a cross sectional study was carried out with the aim to assess the stability of biochemical analytes, being measured in the CNNS survey, by exposing blood to different temperatures and time delay before centrifugation, which may be anticipated in large population studies spanning vast geographical areas.

## Materials and methods

A cross sectional study was designed to assess the stability of biochemical indicators such as haemoglobin, HbA1c and folate in whole blood; ferritin, CRP, vitamin B12, vitamin A (retinol), vitamin D, zinc, sTfR, total cholesterol, HDL, LDL, TG, albumin, total protein and creatinine in serum and glucose in plasma, after exposing blood to different temperature and time periods prior to centrifugation and analysis. To explore ten different combinations of temperature and time and compare it with baseline condition (blood transferred immediately to 2-8 °C after collection and centrifuged after 30 min), a minimum sample size of 35 volunteers *per* group was required to detect a mean percent difference of 0.5 or more between two groups with 80% power and 95% confidence. Mean percent difference of 0.5 was considered in sample size calculation, based on information from prior literature (*3*, *6*).

### Study subjects

Subjects were recruited using a camp approach that was organized in two sub-urban locations in the National Capital Territory, India. A total of 420 adults (above 18 years of age), who volunteered and consented to provide blood sample were recruited in the study.

The study was ethically approved by the ethics committee of All India Institute of Medical Sciences (AIIMS), India. Written consent was obtained from all the volunteers after explaining details of the study through a patient information sheet.

### Study protocol

**Figure 1:**
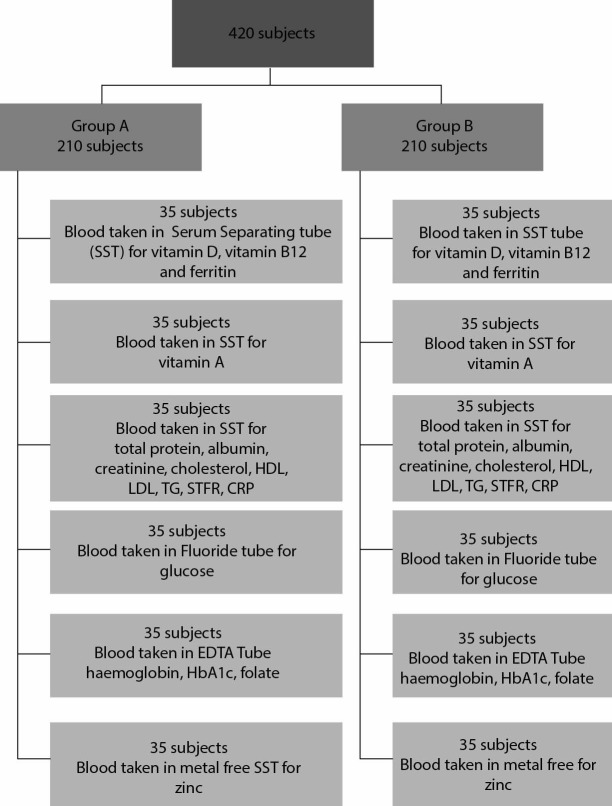
Flowchart showing details of blood sample collected from all recruited subjects, their distribution into different types of tubes and biochemical analytes tested in samples collected in each type of tube. Three set of SST tubes were taken as most analytes required measurement in serum. HDL - high density lipoprotein cholesterol. LDL - low density lipoprotein cholesterol. TG -triglycerides. sTfRC- soluble transferrin receptor. CRP - C-reactive protein. HbA1c - glycated haemoglobin.

**Figure 2:**
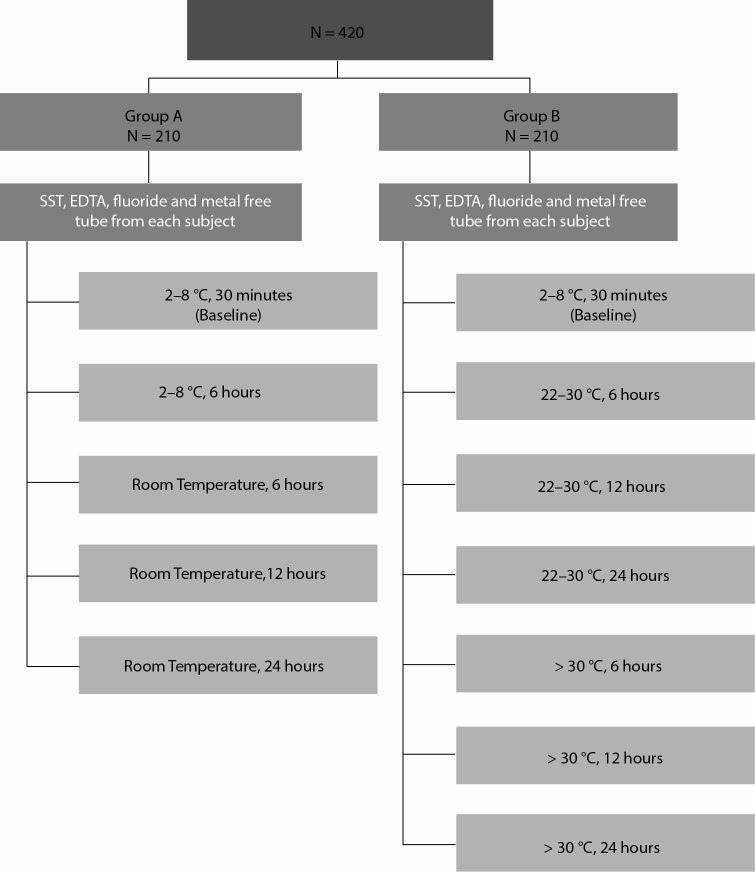
Flowchart showing different temperature and time exposure of blood samples collected from group A and B subjects in various types of tubes. Blood collected in various types of tubes were exposed to different temperatures and time. After exposures serum was separated from SST tubes, plasma was separated from fluoride tube, blood from EDTA tube was used directly for haemoglobin and HbA1c measurement and hemolysate was prepared from EDTA blood for folate measurement.

The study was carried out from December 2017 to January 2018 when the room temperature (RT) was around 18 °C (data logger recorded the RT as 17-19 °C). Venous blood samples were collected by trained phlebotomists from subjects in fasting state. From each subject, 20 mL blood was collected and distributed into either serum separating tube (SST), EDTA or fluoride vacutainers as *per* protocol given in Figure 1. To assess HbA1c, haemoglobin and folate, blood from 70 subjects were collected in EDTA vacutainers (Becton Dickinson, USA). Of the 70 tubes, 35 which constituted group A were exposed to one set of temperature and time (2-8 °C, 30 min and 6 hours and room temperature for 6, 12 and 24 hours) and the other 35 which constituted group B were exposed to different set of temperature and time conditions (2-8 °C, 30 min, 22-30 °C for 6, 12 and 24 hours and > 30 °C for 6, 12 and 24 hours) as outline in Figure 2. For assessing stability of ferritin, vitamin B12 and vitamin D, blood samples from 70 subjects were collected in SST vacutainers (Becton Dickinson, USA) with 35 tubes processed as group A and 35 as group B. Similarly for sTfR, total cholesterol, HDL, LDL, TG albumin, total protein and creatinine, blood samples from 70 subjects were collected into SST vacutainers (Becton Dickinson, USA) and distributed into group A and B. For vitamin A, blood samples from 70 subjects were collected into SST vacutainer; for glucose blood samples from 70 subjects were collected in fluoride vacutainers (Becton Dickinson, USA) and for zinc blood samples from 70 subjects were collected in metal free SST vacutainers (Becton Dickinson, USA) with 35 subjects forming each group. Collection of blood from 70 subjects for each analyte was necessitated as volunteers were unwilling to give 35 mL of blood which was needed to assess all exposures. Since two groups of individuals were engaged for different exposures, we included a baseline condition in both groups which became a reference for that group.

For maintaining 2-8 °C, samples were kept in ice packs inside thermacol boxes. To simulate temperature of 22-30 °C and > 30 °C, hot water bags were used, which were kept in a thermacol box, layered with a cloth towel over which thermacol stands with samples were kept. Data loggers (tempmate ^R^M1, Germany, temperature range - 30 °C to + 70 °C) were kept inside the thermacol boxes to monitor temperature in real time and recorded temperature between 3-6 °C for samples kept in ice packs, 17-19 °C for samples kept at room temperature, 24-26 °C for samples kept at 22-30 °C and 38-40 °C for samples kept at > 30 °C. After bringing the samples to the laboratory, they were shifted to incubators to maintain different temperatures.

Precaution was taken to prevent exposure of samples to light for vitamin A estimation. After different exposures the SST and fluoride tubes were centrifuged at 1700xg for 10 min and serum/plasma samples were separated and stored at - 70 °C till analysis. For erythrocyte folate estimations, haemolysates were prepared by mixing 50 microliters of blood from EDTA tube, immediately after collection, with 1 mL of folate ascorbic acid (Siemens, USA) and subjected to different exposures. For zinc and vitamin A analysis, samples, after exposure and serum separation were transported to National Institute of Nutrition (NIN), Hyderabad, India in dry ice for estimation.

The sample types, principles, methods and the platforms used for estimations of the biochemical analytes are described in Table 1. The methods and platforms were carefully chosen to match that used in the CNNS. Except for vitamin A and zinc analysis which was carried out at National Institute of Nutrition, Hyderabad, all the other analytes were measured at Department of Cardiac Biochemistry, AIIMS, New Delhi. The frozen samples were left at room temperature to thaw before analysis; the aliquots were analysed together in one batch to avoid run to run variability. For vitamin A and zinc, the analysis was carried out in batches over different days. Quality of analysis was ensured by running two levels of controls with each batch of 20 samples. The analytical variation (CVa) for each analyte was computed from the quality control samples assayed over last two months and is reported in Table 2. The AIIMS laboratory participates in RIQAS (RANDOX external quality assurance scheme) EQAS program for all the parameters and NIN participates in VITAL-EQA programme of CDC, Atlanta.

**Table 1:** Details of the biochemical analytes studied for stability in blood sample following exposures to different temperature and time conditions, their methodology for analysis and the platforms / instruments used

**Analyte studied, sample type used**	**Methodology used for analysis**	**Platforms used in analysis**
Haemoglobin, whole blood	Photometric measurement, Cyanmethemoglobin method	3 Part Coulter, Siemens, USA
C-reactive protein, serum	Immunoturbidimetric	Beckman Coulter, AU 480,USA
Total protein, serum	Spectrophotometric, Biuret	Beckman Coulter, AU 680, USA
Albumin, serum	Spectrophotometric, BCP - Dye Binding	Beckman Coulter, AU 680, USA
Ferritin, serum	Chemiluminescence /Two-SiteSandwich Immunoassay	Advia Centaur XP, Siemens, USA
Soluble transferrin receptor, serum	Immunoturbidimetric	Beckman Coulter, AU 480, USA
Vitamin A (Retinol), serum	HPLC-reverse phase chromatography	Thermo Finnigan (spectra system)
Zinc, serum	Atomic absorption spectrometry+F18:F28,	Shimadzu (AA 7000)
Folate, haemolysate	Chemiluminescence / Two-SiteSandwich Immunoassay	Advia Centaur XP, Siemens, USA
Vitamin B12, serum	Chemiluminescence / Two-SiteSandwich Immunoassay	Advia Centaur XP, Siemens, USA
Vitamin 25 (OH) D, serum	Chemiluminescence / Two-SiteSandwich Immunoassay	Advia Centaur XP, Siemens, USA
Glucose, plasma	Spectrophotometric, Hexokinase (UV)	Beckman Coulter, AU 680, USA
Glycylated haemoglobin (HbA1C), whole blood	Ion Exchange HPLC	Variant, Biorad, USA
Total Cholesterol, serum	Spectrophotometric, Cholesterol Oxidase Esterase Peroxidase	Beckman Coulter, AU 680, USA
High Density Lipoprotein Cholesterol (HDL), serum	Spectrophotometric, Direct Measure-Peg/ CHOD	Beckman Coulter, AU 680, USA
Low Density Lipoprotein Cholesterol (LDL), serum	Spectrophotometric, Direct Measure/ CHOD	Beckman Coulter, AU 680, USA
Triglycerides (TG), serum	Spectrophotometric, Enzymatic Endpoint	Beckman Coulter, AU 680, USA
Creatinine, serum	Spectrophotometric, Alkaline Picrate Kinetic (Jaffe’s method)	Beckman Coulter, AU 680, USA

**Table 2:** Analytical coefficient of variation, within subject biological coefficient of variation and total change limit of different biochemical analytes

**Analyte**	**Coefficient of variation, CVa%**	**Coefficient of variation, CVb%**	**Total change limit (TCL)**
HDL- cholesterol	3.7	7.3	± 10.9
Albumin	2.3	3.2	± 6.6
Cholesterol	4.2	5.95	± 12.1
Creatinine	1.8	5.95	± 5.9
Glucose	1.8	4.5	± 5.6
LDL- cholesterol	3.5	7.8	± 10.4
Total Protein	1.0	2.75	± 3.0
Triglyceride	3.5	19.9	± 13.8
CRP	2.6	42.2	± 22.3
Ferritin	10.4	14.2	± 29.6
Folate	6.9	24.0	± 22.6
Vitamin B12	8.8	15.0	± 25.5
Vitamin D	4.8	11.3	± 14.3
HbA1c	1.2	1.9	± 3.5
Haemoglobin	1.5	2.85	± 4.4
Zinc	6.9	9.4	± 19.7
Vitamin A	3.5	19.0	± 13.6
sTfR	3.0	3.0	± 8.4
HDL - high density lipoprotein. LDL - low density lipoprotein. CRP - C reactive protein. HbA1c - glycylated haemoglobin. sTfR - soluble transferrin receptor. CV_b_ - within-subject biological coefficient of variation, obtained from Ricos (*8*), except for Vitamin A and Zinc for which between-subject biological coefficient variation was used. CVa% - analytical coefficient of variation. TCL - total change limit and is an absolute value derived from CVa and CVb using the formula √(2.77 CVa)^2^ + (0.5 CVb)^2^.			

### Statistical Analysis

The percentage deviation in the analyte value was computed by subtracting values at baseline (To) from that at other time periods (Tx) using the following formula:

Percentage deviation = [(Tx − To) / To] × 100

For assessing changes resulting from instability of an analyte, in the same sample from the same individual, analytical variance or coefficient of variation (CVa) and biological coefficient of variation (CVb) (within- subject biological variation) for each biological indicator was taken into consideration. As in this study, the specimens were collected by a single-phlebotomy and then drawn into different vacutainers, biological variation could not be estimated from the study data and therefore CVb for each analyte was taken from the listing of biological variation for 316 analytes by Ricos *et al.* (*8*). The analytical and intra-individual imprecisions were used to estimate the total change limit (TCL) for each analyte in the following manner (*6*).

TCL= √(2.77 CVa)^2^ + (0.5 CVb)^2^

The factor 2.77 is derived from Z√2, where Z = 1.96, determined by the 95% confidence interval value for bi-directional changes. According to the recommendations of the College of American Pathologist the imprecision of a method, for individual single and multipoint testing, should be equal or less than one-half of the average within-subject variation (CVb), and this should be the goal for short-term laboratory imprecision (≤ 0.5 CVb) (*9*, *10*). Table 2 presents the analytical and biological CV% for the different analytes and the computed TCL values.

The mean % deviation of the analytes at each temperature and duration of exposure was compared with the TCL. Values that fell within the TCL were deemed to be stable. In cases where the results for an analyte had a mean percentage difference that exceeded the TCL, then the difference was judged to be significant and not to meet the stability criteria. All statistical analysis was performed using STATA 15.1 (STATAcorp LP, College station, TX 77845, USA).

## Result

**Figure 3:**
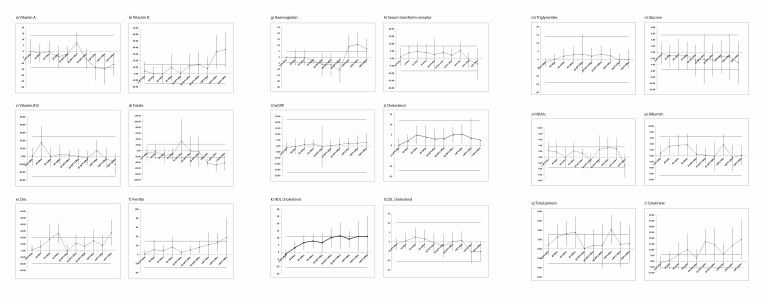
Mean % deviation in biochemical analytes at different temperatures and time duration of exposure prior to analysis. Figure 3 (a-r) shows the mean % deviation of biochemical analytes from baseline values in blood samples exposed to different temperatures (2-8 °C, RT, 22-30 °C and > 30 °C) for varying lengths of time (6 h, 12 h and 24 h) prior to centrifugation. In each figure, the horizontal lines above and below the mean indicate the TCL values.

Figure 3. (a-r) shows the mean % deviation for biochemical analytes, from baseline values, in blood samples exposed to different temperatures and centrifugation time. A mean percentage deviation in vitamin A values beyond TCL was noted at temperature greater than 30 °C and time duration of more than 12 h (Figure 3a). Mean % deviation for vitamin D (Figure 3b), higher than TCL, was reported at temperature greater than 30 °C for 12 hours or more. Deviation in vitamin B12 values was observed to be within the TCL value for all temperature and time periods of exposure (Figure 3c). Mean % deviation was more than TCL when samples were exposed to greater than 30 °C, even for 6 h, for folate (Figure 3d). Zinc values deviated from baseline when not centrifuged for 24 hours at RT or kept at greater than 30 °C (Figure 3e).

Deviation in ferritin levels were found to be within TCL for all temperatures except when exposed to > 30 °C for more than 24 h (an increase observed) (Figure 3f). As evident from figure 3g mean% deviations for haemoglobin values were beyond TCL even when exposed for 12 hours at 22-30 °C. At temperatures more than 30 °C significant haemolysis was noted in the samples and the values were beyond TCL on the higher side even when exposed for 6 h. Deviation in serum transferrin was within TCL limits (Figure 3h). Serum CRP also did not show any significant deviation from baseline at any temperature and duration of exposure (Figure 3i).

As evident from figure 3j, 3k, and 3m, mean % deviation for total cholesterol, LDL cholesterol and triglycerides were found to be within the TCL limits at all temperatures and time period studied. Percentage deviation for HDL values tended to be higher than TCL limits when exposed to high temperatures for more than 12 h (Figure 3l). Deviation in glucose, HbA1c and albumin values were within TCL (Figure 3n-3p). Deviation in protein did not show any clear trend (Figure 3q). Creatinine values deviated significantly when exposed to higher temperatures as well as when centrifugation was delayed (Figure 3r).

## Discussion

We assessed the stability of analytes in blood, that were collected in the field in community settings, and transported and kept at different temperatures (2-8 °C, RT, 22-30 °C and > 30 °C) for varying lengths of time (6 h, 12 h and 24 h) prior to centrifugation, to mimic a survey scenario. Of the 18 analytes studied, vitamin B12, hsCRP, serum transferrin receptor, total cholesterol, LDL cholesterol, triglycerides, glucose, HbA1c and albumin showed stability at all temperatures even when kept uncentrifuged for 24 h. Vitamin A, folate and haemoglobin levels dropped below the TCL when exposed to higher temperatures for longer time periods. Vitamin D, zinc, ferritin and creatinine levels increased with higher temperatures and delays in centrifugation. Creatinine was particularly affected even when exposed to room temperature for 24 h.

Tanner *et al.* assessed the stability of analytes when exposed to 15 °C, 25 °C and 35 °C for 4, 12 and 24 h prior to centrifugation and have reported decrease in glucose and increase in creatinine. We found glucose to be stable in our study but we used plasma separated from fluoride vacutainers containing a glycolytic inhibitor whereas serum was used in the study by Tanner *et al.* (*4*). Creatinine was found to be unstable in our study also. Other authors have also reported a significant increase in measured creatinine if there was a 24 h delay in centrifugation of whole blood. Shepherd *et al.* studied the stability of blood at 21 °C kept uncentrifuged for 15 min, 4 hours (h), 8 h,14 h, 24 h and 31h and found stability only up to 14 h (*11*). The authors reported that, if measured by enzymatic method, the creatinine levels are not affected. Spithoven *et al.* reported stability for creatinine even after delayed centrifugation when assessed by enzymatic method (*12*). Jaffe’s method which is the most commonly used method for creatinine estimation is subject to interference from ketones and pyruvate levels which increases in stored sample. Ferritin was also reported to be unstable at higher temperature by Tanner *et al.*, a similar observation to that reported in our study (*4*).

Ono *et al*. exposed whole blood to higher temperature (> 30 °C) for up to 48h and found no significant changes in albumin, zinc, total cholesterol, triglyceride and creatinine which is similar to our observations (*13*). Significant changes for total protein after 48h at 30 °C was also reported in that study. Glucose was analysed in the serum and found to be highly unstable. For glucose estimation vacutainers with sodium fluoride as an additive is recommended and other authors have also reported its stability up to 24 h in whole blood stored at temperatures ranging from 4 °C to 40 °C (*14*).

The results observed in this study are consistent with that reported in a meta-analysis of several stability studies published by World Health Organization (*15*). The WHO publication has reported stability up to 25 °C for most analytes mentioned in our study, the difference in our study being that we have also evaluated stability at higher temperature which mimics the conditions during summer months in India.

Studies conducted by Hankinson *et al.* as an off shoot of Nurses’ Health Study and Heins *et al.* have investigated the effect of storage of whole blood at ambient temperature (RT) or cooler temperature (8-9 °C) up to 72 h, the Nurse Health Study also stimulated the transport conditions to see its effect (*16*, *17*). The authors looked at lipids and vitamins in these studies and reported a variation, though not statistically significant, for HDL-cholesterol, β-carotene and retinol in samples that was kept at RT and transported to mimic the field conditions. Among the other biochemical parameter studied by Hans *et al.* an increase in the concentration of creatinine and HDL when kept at RT for prolonged time *i.e.* beyond 24h was reported. In our study the mean difference of HDL was within the TCL when kept at RT, however at higher temperature the difference was outside the TCL even with 6 hours of storage.

Oddoze *et al.* reported stability of total protein, albumin, creatinine, total cholesterol, triglyceride, HDL and LDL cholesterol, CRP, soluble transferrin receptor and HbA1c at RT (25 °C) even if centrifuged after 24 h. The stability of vitamin D and vitamin B12 extended to 72 h in whole blood; folate was however found to be unstable (*6*). Clark *et al.* have also shown stability for biochemical analytes in whole blood samples kept at room temperature for a week before separation however creatinine was unstable, as shown in our study (*18*).

In a hospital based study conducted by Henriksen *et al.* feasibility of analysing blood samples for biochemical and immunological analytes when stored for 10h at 21°C prior to separation was explored (*3*). They did not find any statistically significant bias for albumin and creatinine; a small bias was observed for cholesterol, CRP, ferritin, triglyceride and a more pronounced bias for B12, folate and HDL-c but none of the bias exceeded the goal envisaged for that study (*3*). The level of folate decreased which is similar to the observation of this study. The folate was outside TCL at higher temperature even beyond 6hr of storage. Folate is highly unstable and susceptible to oxidative cleavage (*19*, *20*). A study that has looked at storage of several analytes up to a maximum of 96 h at 21°C CRP, retinol, and ferritin was reported to be stable; the mean concentration of folic acid however changed significantly over time, with the sharpest decrease (8.7%) in the first 2 h of storage (*21*). Vitamins A, E, K, B1, B2, B12, RBC folate and carotenoids were shown to be stable in whole blood when stored refrigerated or at RT even up to 48 or 72 h. Serum folic acid showed clinically and statistically signiﬁcant changes in mean concentration after delayed centrifugation (*22*).

Many studies have shown stability of haemoglobin and its components even up to 72 hr at 4°C and room temperature, but stability of haemoglobin at higher temperatures have not been documented (*23*, *24*). We found a significant change in haemoglobin values at above 25 °C even at 6h. Hence maintaining a cold chain for blood samples collected for haematological parameters is highly imperative in surveys and epidemiological studies.

Based on our study we can say that in community surveys blood samples for creatinine, haemoglobin and folate should not be exposed to high temperatures (> 30 °C) and should be processed within 12 hours of collection for reliable reports. Albumin, CRP, glucose, cholesterol, LDL, TG, vitamin B12 and A, sTfR and HbA1c can be reliably estimated even in blood samples exposed to high temperature (> 30 °C) and centrifuged after 12 hours in surveys.

Several factors can affect the analytes due to delay in centrifugation or exposure to high temperature. Exchange between serum and erythrocyte continues in uncentrifuged blood samples resulting in either an increase in analytes or a dilution of analytes. Cell lysis due to exposure at high temperature will also lead to release of intracellular content resulting in increase in some analytes. Haemoglobin released due to haemolysis can interfere with some photometric assays. Bacterial growth with prolonged storage at higher temperature can also affect analyte concentrations. Increase/decrease in analyte concentrations observed in the present study could be explained by these factors.

In most of the above-mentioned studies, different aspects of stability such as different samples, different temperatures, various exposure times and different tubes were investigated and the sample size was around 10-15. The strength of our study was firstly that we used a large sample size to study each temperature and time exposure. We also studied exposure at higher temperatures which is encountered in summer months in several countries. Most importantly we also mimicked survey conditions by collecting samples in the field and incorporating a transportation component which is an important consideration not highlighted in many studies.

The limitation of our study was that we were not able to motivate volunteers to provide 30-35 mL of blood which would have allowed us to study all exposures in the same individual. To overcome this we engaged two groups for studying all the exposures and included baseline (reference) measurement in both groups.

In conclusion, among the analytes studied by us, all analytes other than creatinine, folate and haemoglobin could be reliably estimated in blood samples exposed to 22 – 30 °C for 12 hours. Our findings indicate that in community based surveys, a delay of up to 12 hours between the collection of a blood sample and centrifugation seems to be acceptable to provide valid results for biochemical analytes reported in this study. These findings are encouraging and will serve to facilitate the extension of the reach of laboratory services to the remote corners of the country.
